# Multi-target-qubit unconventional geometric phase gate in a multi-cavity system

**DOI:** 10.1038/srep21562

**Published:** 2016-02-22

**Authors:** Tong Liu, Xiao-Zhi Cao, Qi-Ping Su, Shao-Jie Xiong, Chui-Ping Yang

**Affiliations:** 1 Department of Physics, Hangzhou Normal University, Hangzhou, Zhejiang 310036, China

## Abstract

Cavity-based large scale quantum information processing (QIP) may involve multiple cavities and require performing various quantum logic operations on qubits distributed in different cavities. Geometric-phase-based quantum computing has drawn much attention recently, which offers advantages against inaccuracies and local fluctuations. In addition, multiqubit gates are particularly appealing and play important roles in QIP. We here present a simple and efficient scheme for realizing a multi-target-qubit unconventional geometric phase gate in a multi-cavity system. This multiqubit phase gate has a common control qubit but different target qubits distributed in different cavities, which can be achieved using a single-step operation. The gate operation time is independent of the number of qubits and only two levels for each qubit are needed. This multiqubit gate is generic, e.g., by performing single-qubit operations, it can be converted into two types of significant multi-target-qubit phase gates useful in QIP. The proposal is quite general, which can be used to accomplish the same task for a general type of qubits such as atoms, NV centers, quantum dots, and superconducting qubits.

Multiqubit gates are particularly appealing and have been considered as an attractive building block for quantum information processing (QIP). In parallel to Shor algorithm^1^, Grover/Long algorithm^2^,^3^, quantum simulations, such as analogue quantum simulation^4^ and digital quantum simulation^5^, are also important QIP tasks where controlled quantum gates play important roles. There exist two kinds of significant multiqubit gates, i.e., multiqubit gates with multiple control qubits acting on a single target qubit^6^,^7^,^8^,^9^,^10^,^11^,^12^,^13^,^14^, and multiqubit gates with a single qubit simultaneously controlling multiple target qubits^15^,^16^,^17^. These two kinds of multiqubit gates have many applications in QIP such as quantum algorithms^1^,^18^,^19^,^20^, quantum Fourier transform^19^, error correction^21^,^22^,^23^, quantum cloning^24^, and entanglement preparation^25^.

A multiqubit gate can in principle be constructed by using single-qubit and two-qubit basic gates. However, when using the conventional gate-decomposition protocols to construct a multiqubit gate^26^,^27^,^28^, the number of basic gates increases and the procedure usually becomes complicated as the number of qubits increases. Hence, building a multiqubit gate may become very difficult since each basic gate requires turning on and off a given Hamiltonian for a certain period of time, and each additional basic gate adds experimental complications and the possibility of more errors. Thus, the study of reducing the operation time and the number of switching Hamiltonians is crucial in multiqubit gates^29^,^30^,^31^. Proposals have been presented for directly realizing both multi-control-qubit gates^6^,^7^,^8^,^9^,^10^,^11^,^12^,^13^,^14^ and multi-target-qubit gates^15^,^16^,^17^ in various physical systems. However, note that the gate implementation using these previous proposals^6^,^7^,^8^,^9^,^10^,^11^,^12^,^13^,^14^,^15^,^16^,^17^ was based on non-geometric dynamical evolution.

During the past years, there is much interest in fault-tolerant geometric quantum computing based on Abelian geometric phases^32^,^33^,^34^,^35^ and Holonomic quantum computing based on non-Abelian holonomies^36^. The construction of conventional geometric phase gates usually requires to remove the dynamical phase by choosing the adiabatic cyclic evolution^37^ or employing multi-loop schemes (the evolution is driven by a Hamiltonian along several closed loops)^38^,^39^. In recent years, attention has been shifted to unconventional geometric phases introduced in^40^, which can be used as an alternative resource for geometric quantum computation without the need to remove the dynamic phase. According to^40^, an unconventional geometric phase gate is characterized by a unitary operator *U*({*γ*}), where *γ* is the total phase, which consists of a geometric phase and a dynamic phase (see^40^). Thus, *additional* operations are not needed to cancel the dynamical phase, because the total phase is dependent only on global geometric features and independent of initial states of the system. In this paper, we mainly focus on the construction of multiqubit gates based on unconventional geometric phases.

A number of proposals have been presented for realizing both conventional and unconventional geometric phase gates^37^,^38^,^39^,^40^,^41^,^42^,^43^,^44^,^45^,^46^,^47^,^48^,^49^,^50^,^51^. Some approaches also combine the geometric computation with other theories in order to improve the robustness (e.g., combined with decoherence free subspace or dynamical decoupling)^50^,^51^. Moreover, high-fidelity geometric phase gates have been experimentally demonstrated in several physical systems^52^,^53^,^54^,^55^,^56^,^57^. For instances, Jones *et al*.^52^ experimentally demonstrated a conditional Berry phase shift gate using NMR, and Leibfried *et al*.^53^ realized a two-qubit geometric phase gate in a trapped ion system. On the other hand, much progress has been achieved in Holonomic quantum computing. Experimentally, Abdumalikov Jr *et al*.^54^ realized single-qubit Holonomic gates in a superconducting transmon, Feng *et al*.^55^ implemented one-qubit and two-qubit Holonomic gates in a liquid-state NMR quantum information processor, and two groups^56^,^57^ demonstrated single-qubit or two-qubit Holonomic gates using the NV centers at room temperature, respectively. However, we note that previous works mainly focus on constructing single- or two-qubit geometric phase gates/Holonomic gates^37^,^38^,^39^,^40^,^41^,^42^,^43^,^44^,^45^,^46^,^47^,^48^,^49^,^50^,^51^,^52^,^53^,^54^,^55^,^56^,^57^, or implementing a multi-control-qubit gate^6^,^7^,^8^,^9^,^10^,^11^,^12^,^13^,^14^ and a multi-target-qubit gate^15^,^16^,^17^ based on non-geometric dynamical evolution.

In this work, we consider how to implement a multi-target-qubit unconventional geometric phase gate, which is described by the following transformation:
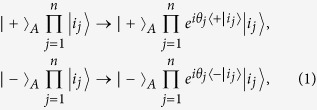
where subscript *A* represents a control qubit, subscripts (1, 2, ..., *n*) represent *n* target qubits (1, 2, ..., *n*), and 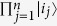 (with *i*_*j*_ ∈ {+, −}) is the *n*-target-qubit computational basis state. For *n* target qubits, there are a total number of 2^*n*^ computational basis states, which form a set of complete orthogonal bases in a 2^*n*^-dimensional Hilbert space of the *n* qubits. Equation (1) shows that when the control qubit *A* is in the state 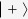 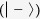, a phase shift 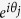 happens to the state 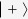 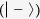 but nothing happens to the state 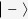 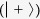 of the target qubit *j* (*j*  = 1, 2, ..., *n*). For instance, under the transformation (1), one has: (i) the state transformation described by following Eq. (18) for a two-qubit phase gate on control qubit *A* and target qubit *j*, and (ii) the state transformation described by Eq. (21) below for a three-qubit phase gate on control qubit *A* and two target qubits (1, 2). Note that the multiqubit phase gate described by Eq. (1) is equivalent to such *n* two-qubit phase gates, i.e., each of them has a common control qubit *A* but a different target qubit 1, 2, ..., or *n*, and the two-qubit phase gate acting on the control qubit *A* and the target qubit *j* (*j* = 1, 2, ..., *n*) is described by Eq. (18).

The multiqubit gate described by Eq. (1) is generic. For example, by performing a single-qubit operation such that 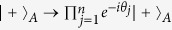 and 
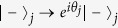
 but nothing to 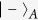 and 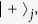 the transformation (1) becomes
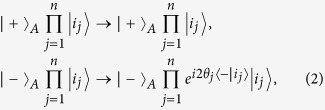
which implies that when and only when the control qubit *A* is in the state 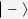, a phase shift 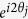 happens to the state 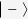 of the target qubit *j* but nothing otherwise (see Fig. 1). For *θ*_*j*_ = *π*/2, the state transformation (2) corresponds to a multi-target-qubit phase gate, i.e., if and only if the control qubit *A* is in the state 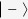, a phase flip from the sign + to − occurs to the state 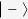 of each target qubit. Note that a CNOT gate of one qubit simultaneously controlling *n* qubits, (see Fig. 1(b) in^15^), can also be achieved using this multiqubit phase gate combined with two Hadamard gates on the control qubit^15^. Such a multiqubit phase or CNOT gate is useful in QIP. For instance, this multiqubit gate is an essential ingredient for implementation of quantum algorithm (e.g., the discrete cosine transform^20^), the gate plays a key role in quantum cloning^24^ and error correction^23^, and it can be used to generate multiqubit entangled states such as Greenberger-Horne-Zeilinger states^25^. This multiqubit gate can be combined with a set of universal single- or two-qubit quantum gates to construct quantum circuits for implementing quantum information processing tasks^20^,^23^,^24^,^25^. In addition, for *θ*_*j*_ = *π*/2^*j*^, the state transformation (2) corresponds to a multi-target-qubit phase gate, i.e., if and only if the control qubit *A* is in the state 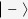, a phase shift *θ*_*j*_ = *π*/2^*j*^ happens to the state 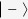 of each target qubit. It is noted that this multi-target-qubit gate is equivalent to a multiqubit gate with different control qubits acting on the same target qubit (see Fig. 2), which is a key element in quantum Fourier transform^1^,^19^.

In what follows, our goal is propose a simple method for implementing a generic unconventional geometric (UG) multi-target-qubit gate described by Eq. (1), with one qubit (qubit *A*) simultaneously controlling *n* target qubits (1, 2, ..., *n*) distributed in *n* cavities (1, 2, ..., *n*). We believe that this work is also of interest from the following point of view. Large-scale QIP usually involves a number of qubits. Placing many qubits in a single cavity may cause some fundamental problems such as introducing the unwanted qubit-qubit interaction, increasing the cavity decay, and decreasing the qubit-cavity coupling strength. In this sense, large-scale QIP may need to place qubits in multiple cavities and thus require performing various quantum logic operations on qubits distributed in different cavities. Hence, it is important and imperative to explore how to realize multiqubit gates performed on qubits that are spatially-separated and distributed in different cavities.

As shown below, this proposal has the following features and advantages: (i) The gate operation time is independent of the number of qubits; (ii) The proposed multi-target-qubit UG phase gate can be implemented using a single-step operation; (iii) Only two levels are needed for each qubit, i.e., no auxiliary levels are used for the state coherent manipulation; (iv) The proposal is quite general and can be applied to accomplish the same task with a general types of qubits such as atoms, superconducting qubits, quantum dots, and NV centers. To the best of our knowledge, this proposal is the first one to demonstrate that a multi-target-qubit UG phase gate described by (1) can be achieved with one qubit simultaneously controlling *n* target qubits distributed in *n* cavities.

In this work we will also discuss possible experimental implementation of our proposal and numerically calculate the operational fidelity for a three-qubit gate, by using a setup of two superconducting transmission line resonators each hosting a transmon qubit and coupled to a coupler transmon qubit. Our numerical simulation shows that highly-fidelity implementation of a three-qubit (i.e., two-target-qubit) UG phase gate by using this proposal is feasible with rapid development of circuit QED technique.

## Results

### Model and Hamiltonian

Consider a system consisting of *n* cavities each hosting a qubit and coupled to a common qubit *A* [Fig. 3(a)]. The coupling and decoupling of each qubit from its cavity can be achieved by prior adjustment of the qubit level spacings. For instance, the level spacings of superconducting qubits can be rapidly adjusted by varying external control parameters (e.g., magnetic flux applied to the superconducting loop of a superconducting phase, transmon, Xmon or flux qubit; see, e.g.^58^,^59^,^60^,^61^); the level spacings of NV centers can be readily adjusted by changing the external magnetic field applied along the crystalline axis of each NV center^62^,^63^; and the level spacings of atoms/quantum dots can be adjusted by changing the voltage on the electrodes around each atom/quantum dot^64^. The two levels of coupler qubit *A* are denoted as 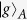 and 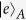 while those of intracavity qubit *j* as 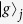 and 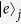 (*j* = 1, 2, ···, *n*). A classical pulse is applied to qubit *A* and each intracavity qubit *j* [Fig. 3(b,c)]. For identical qubits, we have 
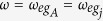
, where *ω* is the pulse frequency and 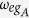 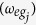 is the 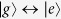 transition frequency of qubit *A* (qubit *j*). The system Hamiltonian in the interaction picture reads (in units of *ħ* = 1)
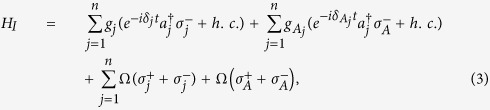
where 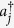 is the photon creation operator for the mode of cavity *j*, 
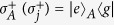
 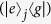 and 
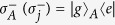
 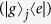 are the raising and lowering operators for qubit *A* (qubit *j*), 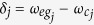 and 
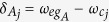
 are detunings (with 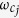 being the frequency of cavity *j*), Ω is the Rabi frequency of the pulse applied to each qubit, 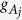 (*g*_*j*_) is the coupling constant of qubit *A* (*j*) with cavity *j*. We choose 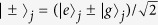 and 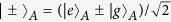 as the rotated basis states of qubit *j* and qubit *A*, respectively.

In a rotated basis 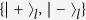, one has 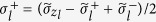 and 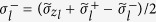, where 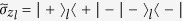, 
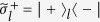
, and 
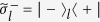
. Here, *l* = 1, 2, 3, ···*n*, *A*. Hence, the Hamiltonian (3) can be expressed as
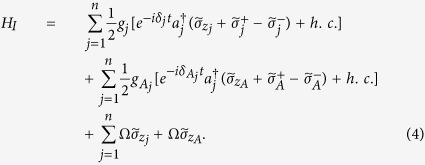
In a new interaction picture under the Hamiltonian 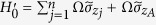, one obtains from Eq. (4)
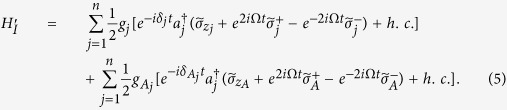
In the strong driving regime 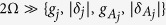, one can apply a rotating-wave approximation and eliminate the terms that oscillate with high frequencies. Thus, the Hamiltonian (5) becomes
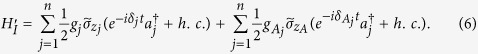
For simplicity, we set
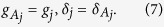
The first term of condition (7) can be achieved by adjusting the position of qubit *j* in cavity *j*, and second term can be met for identical qubits. Thus, the Hamiltonian (6) changes to
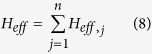
with
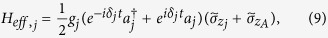
where 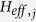 is the effective Hamiltonian of a subsystem, which consists of qubit *A*, intracavity qubit *j*, and cavity *j*. In the next section, we first show how to use the Hamiltonian (9) to construct a two-qubit UG phase gate with qubit *A* controlling the target qubit *j*. We then discuss how to use the effective Hamiltonian (8) to construct a multi-qubit UG phase gate with qubit *A* simultaneously controlling *n* target qubits distributed in *n* cavities.

### Implementing multiqubit UG phase gates

Consider a system consisting of the coupler qubit *A* and an intracavity qubit *j*, for which 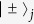 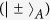 are eigenstates of the operator 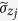 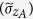 with eigenvalues ±1. In the rotated basis 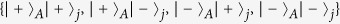, the Hamiltonian (9) can be rewritten as
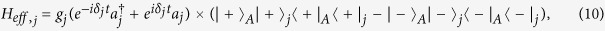
and thus the time evolution operator *U*_*Aj*_(*t*) corresponding to the Hamiltonian 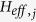 can be expressed as
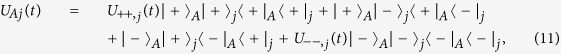
where 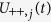 and 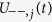 are given by
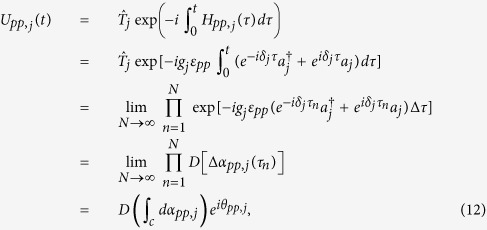
with
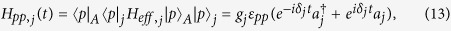
where *pp* ∈ {++, − −}, *p* ∈ {+, −}, *ε*_++_ = −*ε*_−−_ = 1, *D* is the displacement operator (for details, see Methods below), 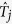 is the time ordering operator and Δ*τ* = *t*/*N* is the time interval. From Eq. (12) and Eq. (31) below, one obtains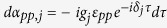 and 
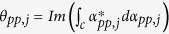
. Thus, one has
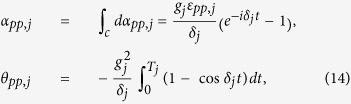
where *T*_*j*_ is the evolution time required to complete a closed path.

If *t* = *T*_*j*_ is equal to 2*m*_*j*_*π*/|*δ*_*j*_| with a positive integer *m*_*j*_, we have ∫_*c*_*α*_*pp*,*j*_ = 0 according to Eq. (14), which shows that when cavity *j* is initially in the vacuum state, then cavity *j* returns to its initial vacuum state after the time evolution completing a closed path. Thus, it follows from Eq. (12) that we have
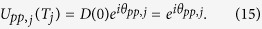
Here *θ*_*pp*,*j*_ is the total phase given by Eq. (14), which is acquired during the time evolution from *t* = 0 to *t* = *T*_*j*_. Note that *θ*_*pp*,*j*_ consists of a geometric phase and a dynamical phase.

It follows from Eqs (11) and (15) that the cyclic evolution is described by
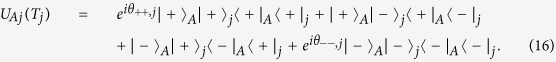
Eq. (14) shows that *θ*_*pp*,*j*_ is independent of index *pp*. Thus, we have *θ*_++,*j*_ = *θ*_−−,*j*_ ≡ *θ*_*j*_. Further, according to Eq. (14), after an integration for *T*_*j*_ = 2*m*_*j*_*π*/|*δ*_*j*_| (set above), we have
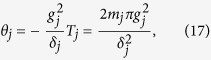
which can be adjusted by varying the coupling strength *g*_*j*_ and detuning *δ*_*j*_. Note that a negative detuning *δ*_*j*_ < 0 [see Fig. 3(b,c)] has applied to the last equality of Eq. (17). The unitary operator (16) describes a two-qubit UG phase gate operation. For *θ*_*j*_ ≠ 2*nπ* with an i*n*teger *n*, the phase gate is nontrivial. After returning to the original interaction picture by performing a unitary transformation 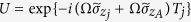, we obtain the following state transformations: 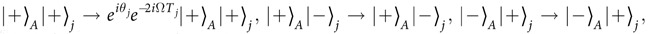 and 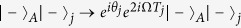, which can be further written as
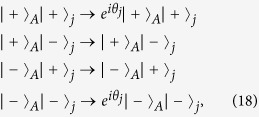
where we have set Ω*T*_*j*_ = *kπ* (*k* is a positive integer). For *T*_*j*_ = 2*m*_*j*_*π*/|*δ*_*j*_|, we have 2Ω = *k*|*δ*_*j*_|/*m*_*j*_. The result (18) shows that a two-qubit UG phase gate was achieved after a single-step operation described above.

Now we expand the above procedure to a multiqubit case. Consider qubit *A* and *n* qubits (1, 2, ···, *n*) distributed in *n* cavities [Fig. 3(a)]. From Eqs (8) and (9), one can see that: (i) each term of *H*_*eff*_ acts on a different intra-cavity qubit but the same coupler qubit *A*, and (ii) any two terms of *H*_*eff*_, corresponding to different *j*, commute with each other: 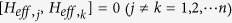. Thus, it is straightforward to show that the cyclic evolution of the cavity-qubit system is described by the following unitary operator
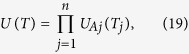
where *U*_*Aj*_(*T*_*j*_) is the unitary operator given in Eq. (16), which characterizes the cyclic evolution of a two-qubit subsystem (i.e., qubit *A* and intracavity qubit *j*) in the rotated basis 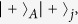 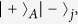 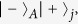 and 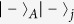.

By changing the detunings *δ*_*j*_ (e.g., via prior design of cavity *j* with an appropriate frequency), one can have
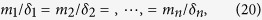
which leads to *T*_1_ = *T*_2_ = , ···, = *T*_*n*_ ≡ *T*, i.e., the evolution time for each of qubit pairs (*A*, 1), (*A*, 2), ···, and (*A*, *n*) to complete a cyclic evolution is identical. For the setting here, we have 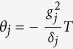 resulting from Eq. (17). Hence, one can easily find from Eqs (18) and (19) that after a common evolution time *T*, the *n* two-qubit UG phase gates characterized by a jointed unitary operator *U*(*T*) of Eq. (19), which have a common control qubit *A* but different target qubits (1, 2, ..., *n*), are simultaneously implemented. As discussed in the introduction, the *n* two-qubit UG phase gates here are equivalent to a multiqubit UG phase gate described by Eq. (1). Hence, after the above operation, the proposed multiqubit UG phase gate is realized with coupler qubit *A* (control qubit) simultaneously controlling *n* target qubits (1, 2, ···, *n*) distributed in *n* cavities.

**Figure 1:**
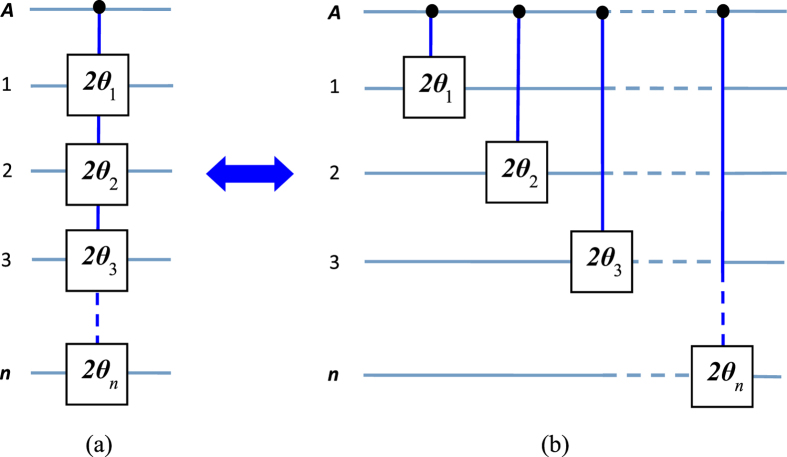
(**a**) Schematic circuit of a phase gate with qubit *A* (a black dot) simultaneously controlling *n* target qubits (squares). (**b**) This multiqubit phase gate illustrated in (**a**) consists of *n* two-qubit phase gates, each having a shared control qubit (qubit *A*) but a different target qubit (qubit 1, 2, ···, or *n*). Here, the element 2*θ*_*j*_ represents a phase shift exp(*i*2*θ*_*j*_), which happens to the state 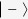 of target qubit *j* (*j* = 1, 2, ..., *n*) when and only when the control qubit *A* is in the state 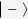 but nothing happens otherwise. For 2*θ*_*j*_ = *π*, this gate corresponds to a multi-target-qubit phase gate (useful in QIP^20^,^23^,^24^,^25^), i.e., if and only if the control qubit *A* is in the state 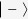, a phase flip from the sign + to − occurs to the state 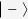 of each target qubit.

**Figure 2:**
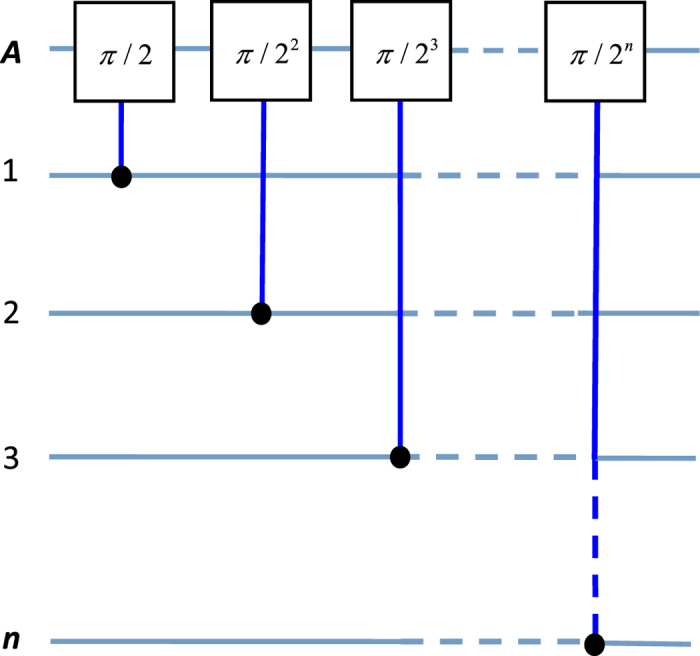
Schematic circuit of the *n* successive two-qubit phase gates in quantum Fourier transform. Here, each two-qubit phase gate has a shared target qubit (qubit *A*) but a different control qubit (qubit 1, 2, ···, or *n*). The element *π*/2^*j*^ represents a phase shift exp(*iπ*/2^*j*^), which happens to the state 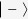 of target qubit *A* if and only if the control qubit *j* is in the state 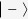 (*j* = 1, 2, ..., *n*). For any two-qubit controlled phase gate described by the transformation 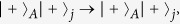 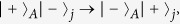 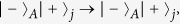 and 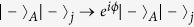, it is clear that the roles of the two qubits can be interchanged. Namely, the first qubit can be either the control qubit or the target qubit, and the same applies to the second qubit. When the second (first) qubit is a control qubit, while the first (second) qubit is a target, the phase of the state 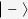 of the first (second) qubit is shifted by *e*^*iϕ*^ when the second (first) qubit is in the state 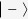, while nothing happens otherwise. Thus, the quantum circuit here is equivalent to the circuit illustrated in Fig. 1 for 2*θ*_*j*_ = *π*/2^*j*^ (*j* = 1, 2, ..., *n*).

**Figure 3:**
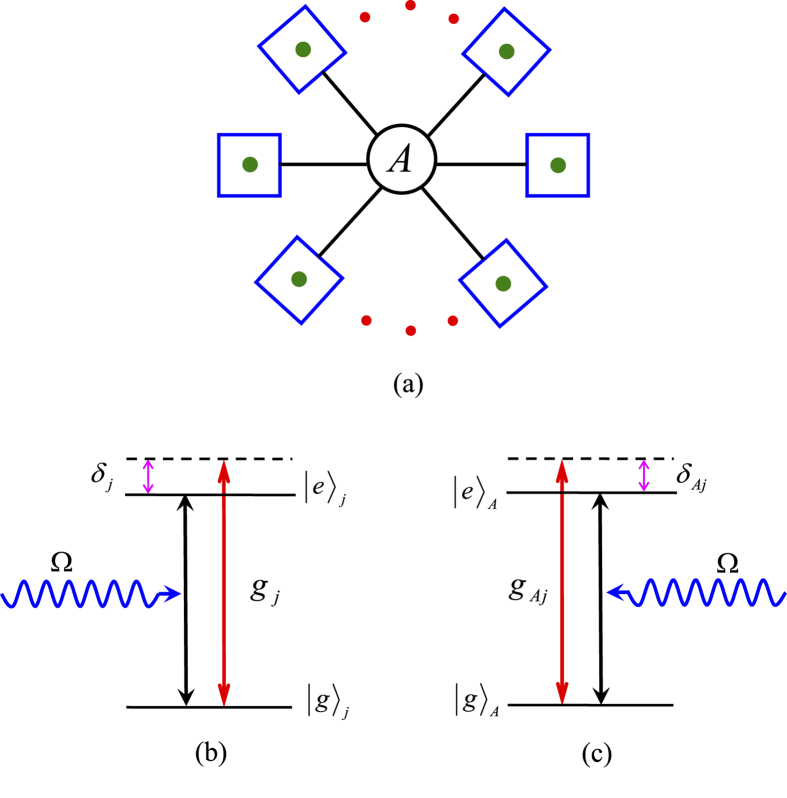
(**a**) Diagram of a coupler qubit *A* and *n* cavities each hosting a qubit. A blue square represents a cavity while a green dot labels a qubit placed in each cavity, which can be an atom or a solid-state qubit. The coupler qubit *A* can be an atom or a quantum dot, and can also be a superconducting qubit capacitively or inductively coupled to each cavity. (**b**) Cavity *j* is dispersively coupled to qubit *j* (placed in cavity *j*) with coupling constant *g*_*j*_ and detuning *δ*_*j*_ < 0. (**c**) The coupler qubit *A* dispersively interacts with cavity *j*, with coupling constant *g*_*Aj*_ and detuning *δ*_*Aj*_ < 0 (*j* = 1, 2, ..., *n*). Here, *δ*_*Aj*_ = *δ*_*j*_, which holds for identical qubits *A* and *j*.

To see the above more clearly, consider implementing a three-qubit (two-target-qubit) UG phase gate. For three qubits, there are a total number of eight computational basis states, denoted by 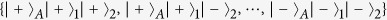. According to Eqs (18) and (19), one can obtain a three-qubit UG phase gate, which is described by
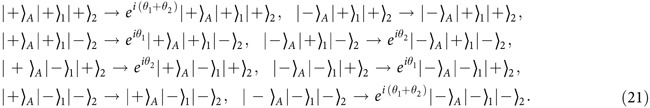
As discussed in the introduction, by applying single-qubit operations, this three-qubit UG phase gate described by Eq. (21) can be converted into a three-qubit phase gate which is illustrated in the above-mentioned Fig. 1 or Fig. 2 for *n* = 2. In the next section, as an example, we will give a discussion on the experimental implementation of this three-qubit UG phase gate for the case of *θ*_1_ = *θ*_2_ = *π*/2. Based on Eq. (17) and for *T*_1_ = *T*_2_ (see above), one can see that the *θ*_1_ = *θ*_2_ corresponds to 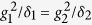, which can be met by adjusting *g*_*j*_ (e.g., varying the position of qubit *j* in cavity *j*) or detuning *δ*_*j*_ (e.g., prior adjustment of the frequency of cavity *j*) (*j* = 1, 2).

### Possible experimental implementation

**Figure 4:**
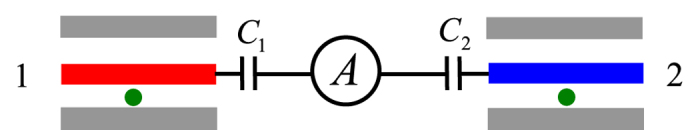
Setup of two cavities (1,2) connected by a superconducting transmon qubit *A*. Here, each cavity represents a one-dimensional coplanar waveguide transmission line resonator, qubit *A* is capacitively coupled to cavity *j* via a capacitance *C*_*j*_ (*j* = 1, 2). The two green dots indicate the two transmon qubits (1, 2) embedded in the two cavities, respectively. The interaction of qubits (1, 2) with their cavities is illustrated in Fig. 5(a,b), respectively. The interaction of qubit *A* with the two cavities is shown in Fig. 5(c). Due to three levels for each qubit considered in our analysis, each qubit is renamed as a qutrit in Fig. 5.

Superconducting qubits are important in QIP due to their ready fabrication, controllability, and potential scalability^58^,^65^,^66^,^67^,^68^,^69^. Circuit QED is analogue of cavity QED with solid-state devices coupled to a microwave cavity on a chip and is considered as one of the most promising candidates for QIP^65^,^66^,^67^,^68^,^69^,^70^,^71^,^72^. In above, a general type of qubit, for both of the intracavity qubits and the coupler qubit, is considered. As an example of experimental implementation, let us now consider each qubit as a superconducting transmon qubit and each cavity as a one-dimensional transmission line resonator (TLR). We consider a setup in Fig. 4 for achieving a three-qubit UG phase gate. To be more realistic, we consider a third higher level 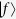 of each transmon qubit during the entire operation because this level 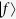 may be excited due to the 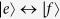 transition induced by the cavity mode(s), which will affect the operation fidelity. From now on, each qubit is renamed “qutrit” since the three levels are considered.

**Figure 5:**
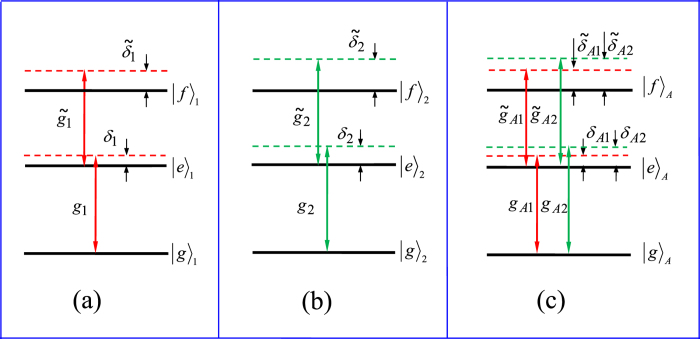
Schematic diagram of qutrit-cavity interaction. (**a**) Cavity 1 is coupled to the 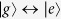 transition with coupling strength *g*_1_ and detuning *δ*_1_, but far-off resonant with the 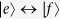 transition of qutrit 1 with coupling strength 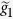 and detuning 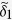. (b) Cavity 2 is coupled to the 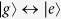 transition with coupling strength *g*_2_ and detuning *δ*_2_, but far-off resonant with the 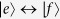 transition of qutrit 2 with coupling strength 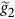 and detuning 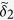. (**c**) Cavity 1 (2) is coupled to the 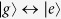 transition of qutrit *A* with coupling strength 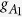 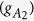 and detuning 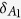 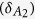; but far-off resonant with the 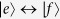 transition of qutrit *A* with coupling strength 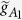 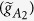 and detuning 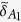 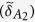. Here, 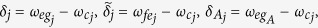 and 
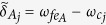
 (*j* = 1, 2), where 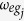 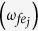 is the 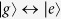 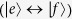 transition frequency of qutrit *j*, 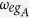 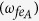 is the 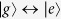 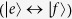 transition frequency of qutrit *A*, and 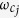 is the frequency of cavity *j*.

When the intercavity crosstalk coupling and the unwanted 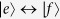 transition of each qutrit are considered, the Hamiltonian (3) is modified as follows
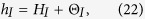
where *H*_*I*_ is the needed interaction Hamiltonian in Eq. (3) for *n* = 2, while Θ_*I*_ is the unwanted interaction Hamiltonian, given by
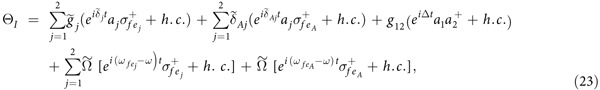
where 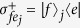 and 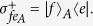 The first term describes the unwanted off-resonant coupling between cavity *j* and the 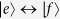 transition of qutrit *j*, with coupling constant 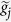 and detuning 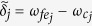 [Fig. 5(a,b)], while the second term is the unwanted off-resonant coupling between cavity *j* and the 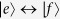 transition of qutrit *A*, with coupling constant 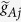 and detuning 
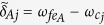
 [Fig. 5(c)]. The third term of Eq. (23) describes the intercavity crosstalk between the two cavities, where 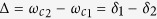 is the detuning between the two-cavity frequencies and *g*_12_ is the intercavity coupling strength between the two cavities. The last two terms of Eq. (23) describe unwanted off-resonant couplings between the pulse and the 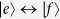 transition of each qutrit, where 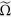 is the pulse Rabi frequency. Note that the Hamiltonian (23) does not involves 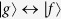 transition of each qutrit, since this transition is negligible because of 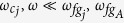 (*j* = 1, 2) (Fig. 5).

When the dissipation and dephasing are included, the dynamics of the lossy system is determined by the following master equation
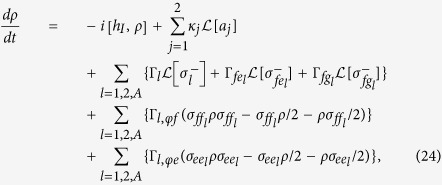
where 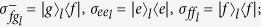 and 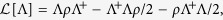 with 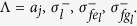 Here, *κ*_*j*_ is the photon decay rate of cavity *j* (*j* = 1, 2). In addition, Γ_*l*_ is the energy relaxation rate of the level 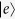 of qutrit *l*, 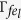 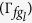 is the energy relaxation rate of the level 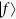 of qutrit *l* for the decay path 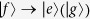, and Γ_*l*,*φ e*_ (Γ_*l*,*φf*_) is the dephasing rate of the level 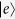 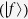 of qutrit *l* (*l* = 1, 2, *A*).

The fidelity of the operation is given by
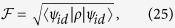
where 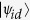 is the output state of an ideal system (i.e., without dissipation, dephasing, and crosstalk considered), while *ρ* is the final density operator of the system when the operation is performed in a realistic physical system. As an example, we consider that qutrit *l* is initially in a superposition state 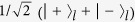 (*l* = 1, 2, *A*) and cavity 1 (2) is initially in the vacuum state. In this case, we have 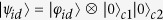, where
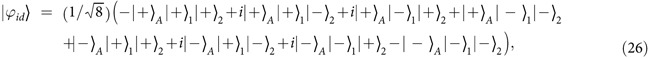
which is obtained based on Eq. (21) and for *θ*_1_ = *θ*_2_ = *π*/2.

We now numerically calculate the fidelity of the gate operation. Without loss of generality, consider identical transmon qutrits and cavities. Setting *m*_1_ = 1 and *m*_2_ = 2, we have *δ*_2_ = 2*δ*_1_ because of Eq. (20), which corresponds to 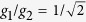 for *θ*_1_ = *θ*_2_. In order to satisfy the relation 2Ω ≫ |*δ*_2_| and 2Ω = *k*|*δ*_2_|/2, we set *k* = 18. In addition, we have 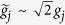, 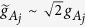 (*j* = 1, 2), and 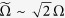 for the transmon qutrits^73^. For a transmon qutrit, the anharmonicity *α*/2*π* = 720 MHZ between the 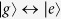 transition frequency and the 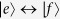 transition frequency is readily achieved in experiments^74^. Thus, we set 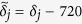 MHz and 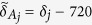 MHz (*j* = 1, 2). For transmon qutrits, the typical transition frequency between two neighbor levels is between 4 and 10 GHz^75^,^76^. Therefore, we choose 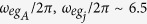 GHz. Other parameters used in the numerical calculation are as follows: 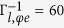 *μ*s, 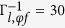 *μ*s, 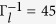 *μ*s, 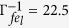 *μ*s, 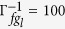 *μ*s (*l* = 1, 2, *A*), and 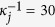 *μ*s (*j* = 1, 2). It is noted that for a transmon qutrit, the 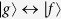 dipole matrix element is much smaller than that of the 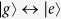 and 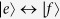 transitions. Thus, 
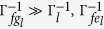
.

**Figure 6:**
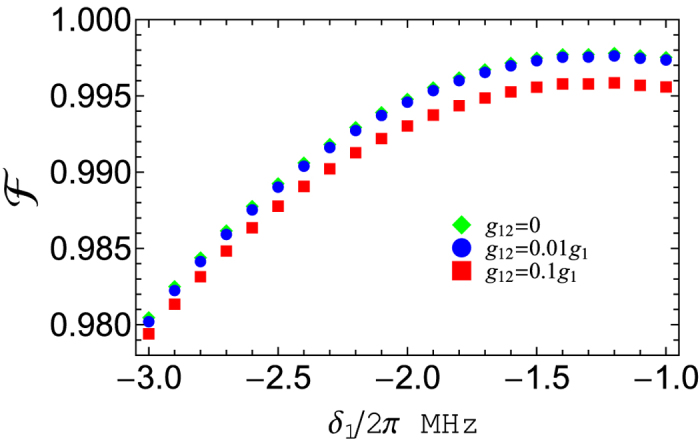
Fidelity versus *δ*_1_/2*π*, plotted for different intercavity coupling strengths but without considering the systematic dissipation and dephasing for simplicity.

To test how the inter-cavity crosstalk affects the gate fidelity, we plot Fig. 6 for *g*_12_ = 0, 0.01*g*_1_, 0.1*g*_1_, which shows the fidelity versus *δ*_1_/2*π*. For simplicity, the dissipation and dephasing of the system are not considered in Fig. 6. As depicted in Fig. 6, the effect of the inter-cavity coupling is negligible as long as *g*_12_ ≤ 0.01*g*_1_.

**Figure 7:**
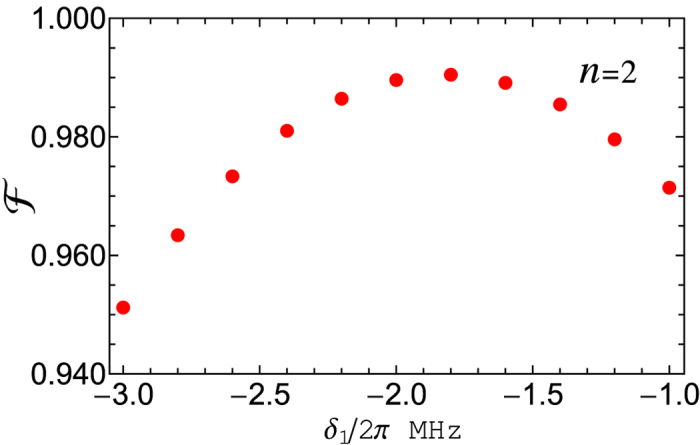
Fidelity versus *δ*_1_/2*π*, plotted for *g*_12_ = 0.01*g*_1_ and by taking the systematic dissipation and dephasing into account. The parameters used in the numerical simulation for Figs 6 and 7 are referred to the text.

Figure 7 shows the fidelity versus *δ*_1_/2*π*, which is plotted by setting *g*_12_ = 0.01*g*_1_ and now taking the systematic dissipation and dephasing into account. From Fig. 7, one can see that for *δ*_1_/2*π* ≈ −1.8 MHz, a high fidelity ~99.1% is achievable for a three-qubit UG phase gate. For *δ*_1_/2*π* ≈ −1.8 MHz, we have *T* = *T*_1_ = *T*_2_ = 0.556 *μ*s, *g*_1_/2*π* = 0.9 MHz, and *g*_2_/2*π* = 1.273 MHz. The values of *g*_1_ and *g*_2_ here are readily available in experiments^77^.

The condition *g*_12_ = 0.01*g*_1_ is easy to satisfy with the cavity-qutrit capacitive coupling shown in Fig. 4. When the cavities are physically well separated, the inter-cavity crosstalk strength is 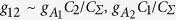, where *C*_Σ_ = *C*_1_ + *C*_2_ + *C*_*q*_ (*C*_*q*_ is the qutrit’s self-capacitance)^78^,^79^. For *C*_1_, *C*_2_~ 1 fF and *C*_Σ_~ 100 fF (typical values in experiments), one has *g*_12_ ~ 0.01*g*_1_. Thus, the condition *g*_12_ = 0.01*g*_1_ is readily achievable in experiments.

Energy relaxation time *T*_1_ and dephasing time *T*_2_ of the level 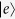 can be made to be on the order of 55–60 *μ*s for state-of-the-art transom devices coupled to a one-dimensional TLR^80^ and the order of 20–80 *μ*s for a transom coupled to a three-dimensional microwave resonator^81^,^82^. For transmon qutrits, we have the energy relaxation time 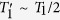 and dephasing time 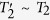 of the level 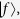 which are comparable to *T*_1_ and *T*_2_, respectively. With 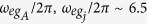 GHz chosen above, we have *ω*_*c*1_/2*π* ~ 6.5018 GHz and *ω*_*c*2_/2*π* ~ 6.5009 GHz. For the cavity frequencies here and the values of 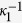 and 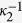 used in the numerical calculation, the required quality factors for the two cavities are *Q*_1_ ~ 1.2249 × 10^6^ and *Q*_2_ ~ 1.2247 × 10^6^. Note that superconducting coplanar waveguide resonators with a loaded quality factor *Q* ~ 10^6^ were experimentally demonstrated^83^,^84^ and planar superconducting resonators with internal quality factors above one million (*Q* > 10^7^) have also been reported^85^. We have numerically simulated a three-qubit circuit QED system, which shows that the high-fidelity implementation of a three-qubit UG phase gate is feasible with rapid development of circuit QED technique.

## Discussion

A simple method has been presented to realize a generic unconventional geometric phase gate of one qubit simultaneously controlling *n* spatially-separated target qubits in circuit QED. As shown above, the gate operation time is independent of the number *n* of qubits. In addition, only a single step of operation is needed and it is unnecessary to employ three-level or four-level qubits and not required to eliminate the dynamical phase, therefore the operation is greatly simplified and the experimental difficulty is significantly reduced. Our numerical simulation shows that highly-fidelity implementation of a two-target-qubit unconventional geometric phase gate by using this proposal is feasible with rapid development of circuit QED technique. The proposed multiqubit gate is generic, which, for example, can be converted into two types of important multi-target-qubit phase gates useful in QIP. This proposal is quite general and can be applied to accomplish the same task with various types of qubits such as atoms, quantum dots, superconducting qubits, and NV centers.

## Methods

### Geometric phase

Geometric phase is induced due to a displacement operator along an arbitrary path in phase space^86^,^87^. The displacement operator is expressed as
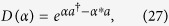
where *a*^†^ and *a* are the creation and annihilation operators of an harmonic oscillator, respectively. The displacement operators satisfy
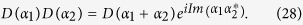
For a path consisting of *N* short straight sections Δ*α*_*j*_, the total operator is
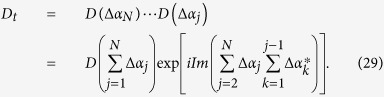
An arbitrary path *c* can be approached in the limit *N* → ∞. Therefore, Eq. (29) can be rewritten as
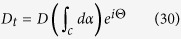
with
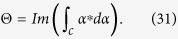
For a closed path, we have
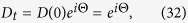
where Θ is the total phase which consists of a geometric phase and a dynamical phase^35^. In above, equations (27, 28, 29, 30, 31, 32) have been adopted for realizing an UG phase gate of one qubit simultaneously controlling *n* target qubits.

## Additional Information

**How to cite this article**: Liu, T. *et al*. Multi-target-qubit unconventional geometric phase gate in a multi-cavity system. *Sci. Rep.* **6**, 21562; doi: 10.1038/srep21562 (2016).
